# Integrated specialty care for amyloidosis: a scoping review using the Consolidated Framework for Implementation Research

**DOI:** 10.1186/s12913-025-12520-3

**Published:** 2025-03-21

**Authors:** Mary O’Sullivan, Wahab Osman, Archanaa Krisnagopal, Monica Parry, Margot Davis, Charlene H. Chu

**Affiliations:** 1 Lawrence Bloomberg Faculty of Nursing, University of Toronto, 155 College St, Toronto, ON M5T 1P8 Canada; 2 Department of Advanced Nursing Practice, School of Nursing and Midwifery, University for Development Studies, Tamale, Ghana; 3 UBC Cardiology, Advanced Heart Failure and Transplant Cardiology, St. Paul’s Hospital, 1081 Burrard Street, Vancouver, BC V6Z 1Y6 Canada; 4 UBC Cardio-Oncology Program and Cardiac Amyloidosis Clinic, Vancouver, Canada; 5 University of Toronto, 155 College St, Toronto, ON M5T 1P8 Canada; 6 Institute for Life Course and Aging, University of Toronto, 155 College St, Toronto, ON M5T 1P8 Canada

**Keywords:** Cardiac amyloidosis, Light chain amyloidosis, Transthyretin amyloidosis, AL amyloidosis, Transthyretin, Multidisciplinary team approach, Clinical pathway, Integrated care, Centre of excellence, Comprehensive care

## Abstract

**Background:**

Amyloidosis is a complex and rare disease requiring specialized, multidisciplinary care to effectively manage its diverse manifestations. Existing evidence underscores the benefits of such care, linked to improved patient outcomes and clinician satisfaction. With the rising incidence of amyloidosis diagnoses and rapid advancements in treatment, the need for coordinated, expert-led care is increasing. However, implementing these centers is challenging due to resource allocation and inter-specialty collaboration. While resource allocation is a known hurdle, there has not been a comprehensive review of all the barriers and facilitators to establishing these clinics. This scoping review aims to identify the barriers and facilitators related to the implementation of coordinated, multidisciplinary specialty care clinics in amyloidosis management.

**Methods:**

An electronic search was conducted in Medline, Embase, and CINAHL for studies published in English from 2013 to 2023, supplemented by a grey literature search. The inclusion criteria focused on studies discussing multidisciplinary clinical environments for amyloidosis care, particularly light-chain (AL) and transthyretin amyloidosis (TTR). Exclusion criteria included books, opinion pieces, dissertations, and conference abstracts. Data were analyzed and synthesized using a narrative synthesis approach, guided by the Consolidated Framework for Implementation Research (CFIR), and reported according to PRISMA-ScR guidelines.

**Results:**

The search resulted in 1547 findings. After screening with Covidence, 7 papers were included in the final review. Independent reviewers screened and extracted the papers. Key facilitators identified include access to experts, adequate staffing, secure funding, partnerships with patient advocacy groups, and robust processes for multidisciplinary communication. Barriers primarily relate to the complexity of care, a lack of standardized protocols, difficulties in communication and coordination between providers, and challenges in training and maintaining knowledgeable care providers. The review also revealed significant gaps in existing research.

**Conclusions:**

This review enhances understanding of the barriers and facilitators in establishing amyloidosis specialty clinics. Addressing these barriers and leveraging facilitators are crucial for shaping the future of amyloidosis care. These insights support a model for implementing integrated care for this growing patient population and highlight the need for further research to support policy development and effective implementation of these specialized clinics.

**Supplementary Information:**

The online version contains supplementary material available at 10.1186/s12913-025-12520-3.

## Introduction and background

### Amyloidosis overview

Amyloidosis is a serious and progressive group of infiltrative diseases characterized by the abnormal folding and deposition of amyloid fibrils in different organs, leading to dysfunction and organ failure [1, 2]. Predominantly (> 98%), amyloidosis manifests in two subtypes: light chain amyloidosis (AL), involving monoclonal immunoglobulin light chains, and transthyretin amyloidosis (TTR), involving transthyretin protein produced by the liver. ATTR amyloidosis can be further categorized into wild-type and familial ATTR amyloidosis [2]. Amyloidosis will refer to AL and ATTR herein. Symptoms and disease severity vary depending on the number of tissue types affected; amyloidosis can impact any organ [3, 4]. The most common and severe manifestations occur with cardiac involvement, called cardiac amyloidosis, with amyloid fibrils depositing in the heart, reducing the heart's ability to pump properly and causing restrictive cardiomyopathy. This can lead to heart failure, arrhythmias, and death [5, 6].

Amyloidosis has historically been considered a rare disease; however, increasing levels of interest and advocacy in this disease have led to an improvement in its recognition, diagnosis, and treatment availability [7]. This shift has changed the trajectory and awareness of amyloidosis care across the globe and has led to an increasing number of confirmed diagnoses [8–10]. Emerging research demonstrates that a significant percentage of patients actually have amyloidosis if they are over 60 years of age with heart failure with preserved ejection fraction (HFpEF), are undergoing valve replacement for aortic valve stenosis, or have a presumed diagnosis of hypertrophic cardiomyopathy [8, 11–14]. The combination of systemic clinical manifestations of this disease and the difficulty of amyloidosis diagnosis, results in high levels of morbidity and mortality [4, 15]. The increasing number of patients being diagnosed and the historically rare nature of amyloidosis diagnosis underscores the importance of awareness and innovation in care in the context of shifting global demographics.

### Complex nature of amyloidosis

AL and TTR amyloidosis pose diagnostic and management challenges due to their multisystem involvement and often non-specific symptoms, which can mimic more common conditions [8, 16, 17]. This complexity, alongside a lengthy and sometimes silent clinical prodrome, contributes to prolonged diagnosis times [17, 18].

Amyloidosis, due to its multisystem nature and predominant impact on the older adult population, is known for its high early mortality rate and extended time to diagnosis, often exceeding one year from symptom onset [4, 8, 17]. As amyloid fibrils can deposit and disrupt multiple organ systems, most AL amyloidosis patients present with involvement of three or more organ systems [17] and those with TTR often have both cardiomyopathy and neuropathy or combinations of multiple symptoms [19]. Therefore, the care of these patients is complex and requires collaboration across multiple specialties and providers [8, 20, 21]. The multidisciplinary involvement often requires but is not limited to, frequent input from cardiology, nephrology, gastroenterology, hematology, neurology, and genetics [8, 20], as well as important roles that nurses and allied health professionals play. The multiorgan involvement and the involvement of multiple specialties confound the complexity of management, as providers working in inherently fragmented healthcare systems will often prescribe care plans and medications that conflict with the care and medications prescribed by other providers [15, 20, 22]. The complexities of medical care can delay diagnosis, cause confusion and anxiety for patients, exacerbate symptoms, and delay crucial interventions, adversely affecting patient outcomes, and contributing to an associated high mortality rate [22–24].

The complexity of care calls for an integrated, coordinated, and multidisciplinary approach that can ensure timely diagnosis and comprehensive management of amyloidosis. Such a model has been shown to improve patient survival and quality of life [8, 20, 25].

### Multidisciplinary clinics

Across the globe, multidisciplinary clinics have significantly improved outcomes for patients with rare and complex diseases, such as sarcoidosis, lung cancer, and neurofibromatosis. Such clinics coordinate comprehensive patient management, integrating diagnosis, treatment, and supportive care, and have set a precedent for the specialized treatment of amyloidosis [26–29].

Amyloidosis, with its diagnostic and treatment complexities, particularly benefits from this expert, coordinated approach. Specialized amyloidosis centres worldwide provide patients with access to a range of integrated services. These centres adopt a patient-centred approach, tailoring care to address not only the medical aspects of amyloidosis but also the educational, psychosocial, and logistical challenges that accompany the disease. Collaboration with other centres, primary care providers, clinical trials, and patient advocacy groups ensures that patients receive comprehensive support throughout their care journey [22, 24, 29–32].

In specialized multidisciplinary care clinics, patients can benefit from core management of their disease or consulting management in collaboration with local care providers [32, 33]. The level of intervention is dependent on the needs of the individuals. Care coordination, frequently overseen by a nurse or nurse practitioner, facilitates efficient collaboration and communication, ensuring that treatment plans are well-suited to meet the particular needs of each patient [24, 32, 34].

Multidisciplinary clinics have been shown to improve outcomes for other complex diseases and are an important part of achieving excellence in amyloidosis care, offering a holistic model that comprehensively addresses the complex needs of patients and drives innovation forward.

### Implementation of multidisciplinary clinics

Even though multidisciplinary clinics are known to help with amyloidosis and other complicated diseases, they are not widely available around the world, making it more challenging for patients to access comprehensive care [19, 23, 32–34]. The low availability of comprehensive specialty care clinics has been attributed to various factors, including the availability of skilled providers, unfavourable reimbursement models, the burden of care coordination, access to funding, and awareness [8, 21, 35].

Given the complexity of implementing multidisciplinary clinics, it is important to consider both the barriers and facilitators to implementation [24, 36]. In the specific context of amyloidosis, there is a gap in the literature concerning the implementation of establishing these clinics. By exploring the barriers and facilitators to the implementation of multidisciplinary amyloidosis clinics, this scoping review aims to map the literature and clearly define the barriers and facilitators experienced in real amyloidosis care centres. These insights could help with further implementation plans, be applicable to other complex medical conditions requiring multidisciplinary care and inform future research in this growing field.

## Methods

### Aim

The aim of this scoping review is to identify and synthesize the barriers and facilitators through the application of the Consolidated Framework for Implementation Research (CFIR) by addressing the research question: What are the barriers and facilitators in implementing multidisciplinary care for amyloidosis patients?

### Design

A scoping review was conducted to understand the barriers and facilitators to implementing multidisciplinary care clinics for amyloidosis. This review approach provides the opportunity to examine and map the available literature and identify key concepts and research gaps, particularly given the scarcity of published literature on the topic [26, 37]. This scoping review methodology was guided by a modified Joanna Briggs Institute (JBI) framework [37]. This framework outlines the following steps: (1) defining and aligning the objective(s) and question(s); (2) developing and aligning the inclusion criteria with the objective(s) and question(s); (3) describing the planned approach to evidence searching, selection, data extraction, and presentation of the evidence; (4) searching for evidence; (5) selecting the evidence; (6) extracting the evidence; (7) analysis of the evidence; (8) presentation of the results, (9) summarizing the evidence in relation to the purpose of the review, making conclusions; and noting any implications. This scoping review followed the PRISMA-ScR (Preferred Reporting Items for Systematic Reviews and Meta-Analyses Extension for Scoping Reviews) checklist (Table S5).

### Theoretical framework

The theoretical framework underpinning the analysis for this review is the updated Consolidated Framework for Implementation Research (CFIR). The CFIR is a widely used framework that facilitates systematic assessment of barriers and facilitators in implementation efforts. The CFIR is composed of five major domains: (1) Intervention Characteristics, which relate to the attributes of the intervention itself; (2) Outer Setting, which includes external influences such as policies and funding; (3) Inner Setting, which references structures, culture, and processes within institutions; (4) Characteristics of Individuals, which relates to the knowledge, beliefs, and engagement of key people involved in implementation; and (5) Implementation Process, which describes the steps taken to plan, execute, and evaluate an intervention. Within these domains, CFIR is further broken down into specific constructs that allow for a nuanced analysis of implementation facilitators and barriers [38]. Operational definitions for this scoping review are found in Table 1. By utilizing CFIR, this review systematically categorizes the factors that impact the successful implementation of multidisciplinary amyloidosis care that can be used to guide systematic assessment of potential barriers and facilitators in implementing coordinated, multidisciplinary care [36].

**Table 1:** Operational definitions of domains and constructs and identification of barriers or facilitators. For each of the coded domain constructs, an operational definition was applied

Domain and Constructs	Operational Definition	Barrier	Facilitator
**Outer Setting**			
Policies and laws	Legislation, regulations, professional group guidelines and recommendations, or accreditation standards support implementation and/or delivery of specialty multidisciplinary amyloidosis clinics	✓	✓
Partnerships and connections	The clinic/centre is networked with external entities, including referral networks, academic affiliations, patient advocacy groups, and professional organization networks	✓	✓
**Inner Setting**			
Structural characteristics	Infrastructure components support functional performance of the clinic/centre. This includes physical infrastructure, information technology, and work infrastructure	✓	✓
Relational connections	There are high quality formal and informal relationships, networks, and teams within and across clinic/centre boundaries		✓
Communications	There are high quality formal and informal information sharing practices within and across clinic/centre boundaries	✓	✓
Tension for change	The current situation of amyloidosis care is intolerable and needs to change		✓
Mission alignment	Implementing and delivering the clinic/centre is in line with the overarching commitment, purpose, or goals of the institution the clinic is located at		✓
Available resources	Resources are available to implement and deliver the implementation of specialized multidisciplinary care	✓	✓
Access to knowledge	Guidance and/or training is accessible to implement and deliver specialized multidisciplinary amyloidosis care	✓	✓
**Implementation Process**			
Planning	Identify roles and responsibilities, outline specific steps and milestones, and define goals for implementation success in advance		✓
Reflecting & evaluating	Collect and discuss quantitative and qualitative information about the success of implementation		✓
**The Innovation**			
Evidence-Base	Specialty multidisciplinary clinics for amyloidosis care has robust evidence supporting their effectiveness		✓
Complexity	The clinic/centre delivery is complicated, which may be reflected by its scope and/or the nature and number of connections and steps related to amyloidosis	✓	✓
**Individuals**			
Innovation beneficiaries (need)	The patients have deficits related to survival, well-being, or personal fulfillment, which will be addressed by implementation and/or delivery of specialized multidisciplinary amyloidosis care		✓
Characteristics (Opportunity)	Individuals who have the availability, scope, and power to fulfill the role in the amyloidoisis clinic	✓	✓

### Search strategy

This scoping review assessed electronically available papers that were published in English between 2013 and October 2023. The search was limited to this timeframe, as care for those diagnosed with amyloidosis has changed considerably over the last ten years due to an increase in diagnostic capabilities, therapeutics, and awareness, resulting in more people seeking and receiving care for amyloidosis [20]. A university health science librarian assisted with the search strategy. An initial search was conducted to confirm search terms and the final search was conducted in three electronic databases: Medline, Embase, and CINAHL. The search was conducted using various synonyms for the following search concepts: (1) amyloidosis and (2) multidisciplinary care. The pilot search strategy was first conducted to ensure search sensitivity, followed by the final search once the search terms were confirmed. The full search strategy for the academic literature is included in Table S1 of the supplementary materials.

The grey literature search took a systematic approach following the University of Toronto’s Grey Literature research guide [39]. This search covered a comprehensive search of government documents, dissertations and theses, policy documents, grey literature databases and targeted web searches and Google searches using keywords “amyloidosis” and “multidisciplinary care”.

The inclusion criteria are: amyloidosis in its two most common forms (amyloid light-chain (AL) and transthyretin (TTR) amyloidosis); and studies that describe either barriers or facilitators to the implementation of a multidisciplinary clinic. The exclusion criteria are books, opinion pieces, dissertations, or conference abstracts. A snowball search of the reference lists from eligible studies was also completed; from this, ten studies were identified; however, they were not included in the final review as they did not meet the eligibility criteria.

### Data screening

Covidence was used to screen papers at both the title/abstract and the full-text level. During screening, MO reviewed abstracts, and MO and WO reviewed full texts independently. Conflicts were resolved between MO and WO through discussion and consensus.

### Data extraction

Data extraction was completed separately for the academic papers and grey literature in Covidence and Microsoft Excel, respectively**.** Study, setting, aim, method, and CFIR characteristics of the academic literature are available in Table S2 of the supplementary materials, and the barriers and facilitators identified by the construct and study are available in Table S4 of the supplementary materials. The grey literature and academic articles were analyzed together to provide a broad overview of the findings. The author, aim, and setting of the grey literature are available in Table S3 of the supplementary materials. In alignment with the Joanna Briggs Institute (JBI) guidelines for scoping reviews, this study did not undertake a quality appraisal of the included studies, as our aim was to summarise the existing research and identify key barriers and facilitators of the implementation of multidisciplinary clinics for amyloidosis, rather than evaluate the rigour of their methods or inform policy [37]. To ensure rigour in the data extraction process, MO and AK extracted data independently, with 43% overlap. MO and AK performed comparison and conflict resolution through discussion and consensus.

## Results

The initial search yielded 1474 findings and 10 references were added from a snowball search. Following the removal of duplicates (351), 1133 papers proceeded to title/abstract screening. 69 studies were reviewed in full-text screening and of these, 7 papers were included in this review.

**Fig. 1:**
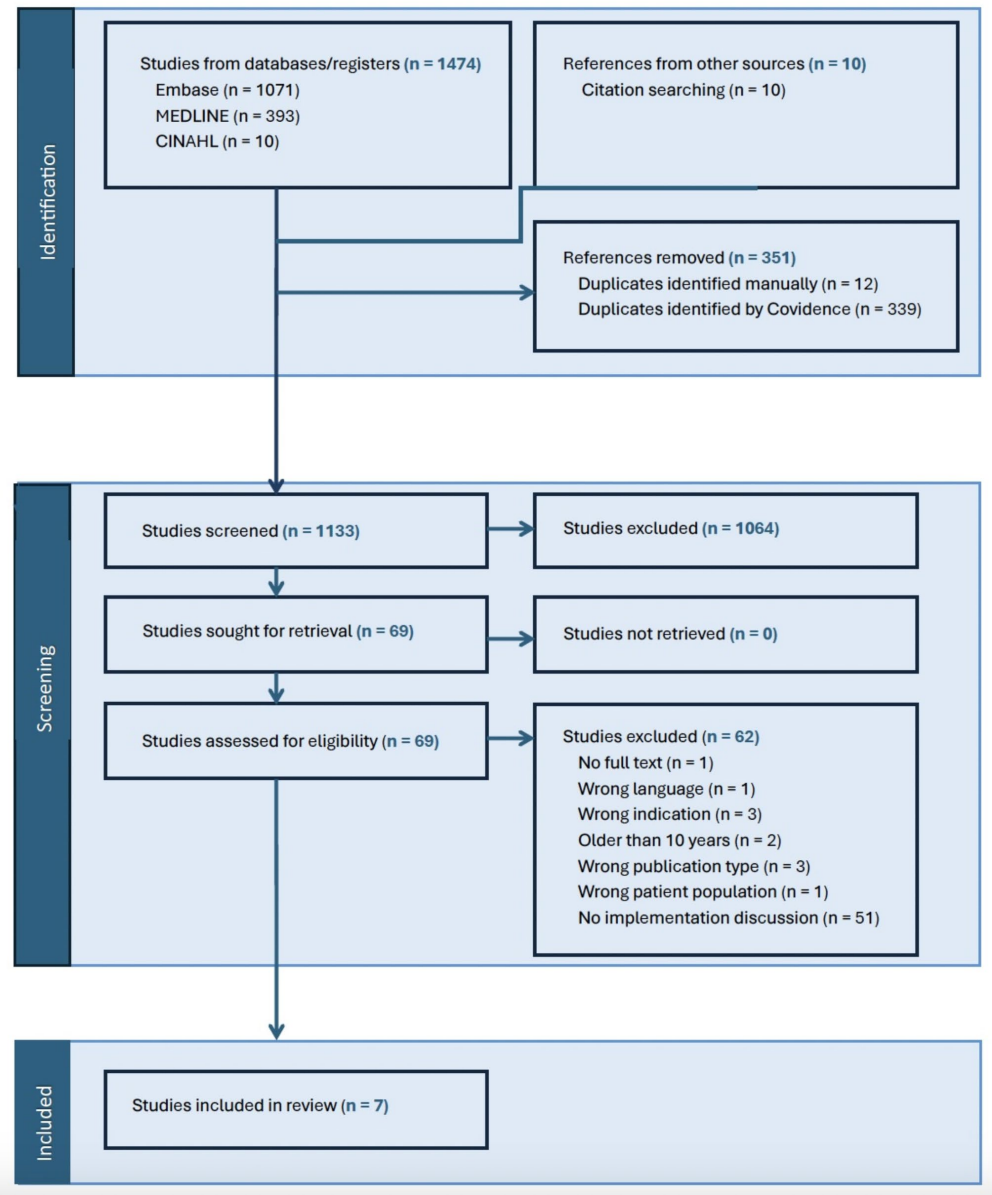
PRISMA flow diagram, which illustrates the academic and grey literature search findings

In the grey literature search, results across all of the Google searches produced 396 results; there were no results found in other sources. Of the Google search results, three were relevant to the implementation of multidisciplinary care in amyloidosis. Two of the grey literature findings were presentations of the findings in two of the articles found; one was a standard contract and description of the amyloidosis service in the National Health Service (NHS) in England. The PRISMA flow diagram outlining the search and findings is represented in Fig. 1.

### Data analysis and presentation

The data analysis employed a deductive thematic approach using the Consolidated Framework for Implementation Research (CFIR) as the guiding framework. The extracted data was analyzed by MO by reviewing the content categorized within the CFIR framework. Structures were identified at the domain level and according to the particular construct within each domain to provide insight into barriers and facilitators for implementation. Due to the lack of an implementation science lens used in the literature regarding multidisciplinary care in amyloidosis, as well as the wide range of terminologies and inconsistent use of language in the field of implementation research, the categorization of data into CFIR constructs was based on the author’s interpretation of definitions and not on definitions made by the study authors. This method ensured a structured and systematic approach to identifying and organizing the barriers and facilitators related to the implementation of multidisciplinary amyloidosis care. The results were synthesized and reported according to the CFIR domains.

### Study characteristics

This review encompassed studies examining multidisciplinary care for amyloidosis in five different countries. Three of the studies were published from the United States of America (USA), one in Spain, one in Bulgaria, one in Greece, and one in Canada.

All included studies were qualitative (*n* = 7, 100%). Four of the studies discussed TTR amyloidosis only (57%); three of the studies discussed both AL and TTR amyloidosis (43%).

**Fig. 2:**
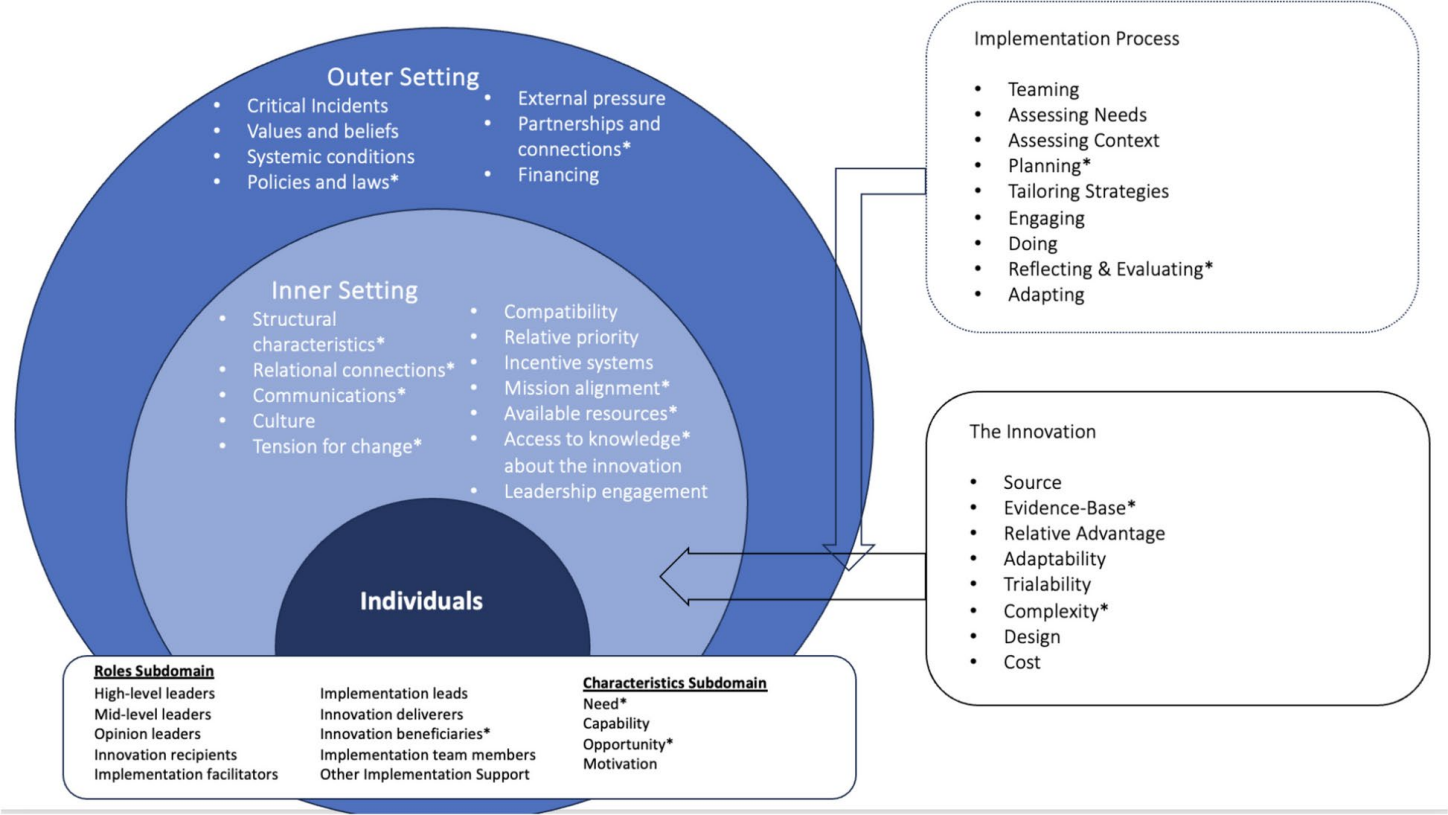
CFIR Model (* = construct discussed in paper)

After the extraction of data, 15 of the 50 (30%) CFIR constructs and their subdomains were coded (coded constructs marked with asterisk in Fig. 2). After reviewing the compiled document, which aggregated all the important barriers and enablers taken from the articles that were included, it was mostly the inner setting where the most important barriers and facilitators were found.

### Facilitators and barriers

A structured summary of the key barriers and facilitators identified in this review is presented in Table 2. Categorized according to CFIR domains, this table highlights the primary factors influencing the implementation of multidisciplinary amyloidosis care, offering a concise reference for researchers and policymakers. Additional details of the barriers and facilitators identified per construct per study can be found in Table S4 of the supplementary materials.

**Table 2:** Summary of barriers and facilitators

CFIR Domain	Key Facilitators	Key Barriers
**Outer Setting**	• Supportive policies and laws around medications and national amyloidosis strategies [23, 26, 40] • Partnerships and connections with other providers, trials, and patient advocacy groups [22–24, 26, 32, 40–42]	• Lack of standardized guidelines and policy barriers [32, 40] • Challenges in coordinating external referrals and diagnostics; limited collaboration between Centres of Excellence [22–24, 32, 41]
**Inner Setting**	• Dedicated funding, institutional support, and specialized multidisciplinary teams [23, 26, 32] • Integration of technology and clear communication pathways for improved care coordination [22, 23, 26, 32, 40]	• Limited institutional funding and resource constraints [22, 24, 40] • Challenges in recruiting and retaining specialized personnel [23, 24, 40]
**Innovation Domain**	• Demonstrated benefits of multidisciplinary amyloidosis care [23, 24, 26]	• Complexity and variability of amyloidosis management [22–24, 40, 41]
**Individual Characteristics**	• Strong leadership and champions • Ongoing education and training programs to enhance provider knowledge [22–24, 26, 32, 40, 41]	• Lack of skilled and trained human resources [22, 24]
**Implementation Process**	• Structured care pathways that include early involvement of key collaborators, training for staff, and partnership creation [22, 40, 41]	• Lack of structured implementation frameworks

A structured summary of the key barriers and facilitators identified in this review is presented in Table 2. Categorized according to CFIR domains, this table highlights the primary factors influencing the implementation of multidisciplinary amyloidosis care, offering a concise reference for researchers and policymakers. Additional details of the barriers and facilitators identified per construct per study can be found in Table S4 of the supplementary materials.

## Outer setting

### Policies and laws

#### Facilitators

Four of the seven articles (57%) reference external policies, laws, and guidelines as facilitators for the establishment of multidisciplinary amyloidosis care. Bumma et al. [26] highlight that the FDA's approval of Tafamidis significantly contributed to the available treatment options that supported the care that multidisciplinary care teams could offer TTR patients [26]. Authors who worked on developing multidisciplinary amyloidosis clinics in Europe (Spain and Greece) cited that the Strategy on Rare Diseases by Spain's Interterritorial Council of the National Health System and the European amyloidosis position statement provide robust frameworks that support the development of specialized units and clinics [23, 40]. In Canada, Davis et al. [24] highlight that best practices for chronic disease management can support the formation of coordinated multidisciplinary care teams in the care of amyloidosis patients.

#### Barriers

In 29% of the articles reviewed (*n* = 2), the absence of standardised guidelines and treatment management was cited as a barrier to effective care for hereditary ATTR amyloidosis. Losada et al. [40] note the global deficiency in management strategies for ATTR and point to legislative frameworks that impede clinical practice, though specifics are not detailed. Furthermore, Nativi-Nicolau [32] reports challenges due to the inconsistency in treatment and surveillance approaches for asymptomatic family members by genetic testing practitioners.

### Partnerships and connections

#### Facilitators

Partnerships and connections were mentioned in all seven (100%) of the articles and in the grey literature as crucial facilitators to the success of implementation. The facilitator aspect can be grouped into six main categories of connections: coordination and referrals, community outreach and education, partnerships, patient advocacy, clinical trial involvement, creation of registries, and data collection [22–24, 26, 32, 40–42].

Coordination between providers enhances the centre's reach, enhances awareness, and increases referrals and integration [22, 24, 26, 40]. Partnerships with national organizations and other centres improve patient care through shared best practices and shared care models [22, 32, 40, 41] and can create opportunities for funding [23]. Engaging with patient advocacy groups and educating stakeholders is crucial for early diagnosis and better outcomes [21, 23, 29, 38], as well as providing support for education for patients and their caregivers [22]. Active research involvement or partnership with centres that are involved in clinical trials facilitates advances in the field and boosts the centres' reputation [24, 26, 32, 40]. Lastly, creating shared patient registries supports clinical pathways and quality improvement [24, 32].

#### Barriers

In 57% (*n* = 4) of the included articles, partnerships and connections are identified as barriers to the implementation of multidisciplinary amyloidosis clinics. Some of the problems mentioned are coordinating care and obtaining diagnostic tests from clinics or providers that refer patients. There is a lack of standardization in the way external referrals are completed and diagnostics are interpreted [22, 24, 32]. Additionally, a clinic in Bulgaria cited a lack of official connections between Centres of Excellence as an issue that hinders collaborative efforts [41].

## Inner setting

The largest number of facilitators and barriers were found within the inner setting.

### Structural characteristics

#### Facilitators

Structural characteristics include physical, IT, and work infrastructure. Within the inner setting, structural characteristics facilitate the functional performance of multidisciplinary amyloidosis care. There were two key areas of structural characteristics that were discussed in the literature: workflow design and physical infrastructure (*n* = 3) and the use of virtual networks and information technology (IT) systems (*n* = 3).

While some clinics reported the benefits of using virtual networks to support their clinic function, a clinic in Ohio that serves as a "one-stop shop" for amyloidosis patients highlights a streamlined workflow design and optimized physical infrastructure by conducting all necessary tests on a particular day and having patients see all necessary providers, with providers rotating between patients [26]. Conversely, the adoption of amyloidosis centres that did not have a distinct physical location or that organized patients to be seen on specific days of the week was highlighted as allowing for more efficient coordination of care [32]. Setting location is likely based on the availability of space, providers, and resources, but it was established by Davis et al. [24] that dedicated clinic space and time would be most optimal for patient care, and that amyloidosis clinics set up within the existing structures of an organization can optimize resource use and integrate with existing institutional care pathways [24].

Additionally, the adoption of technology solutions to support care was noted as essential for handling complex cases, innovation in diagnostics, promoting multidisciplinary communication and care pathways and managing patient education [24]. Moreover, using digital tools and the same electronic medical records across all specialties helps enhance coordination of care, makes administrative tasks easier, and makes it easier to make decisions as a team [22, 23, 32].

#### Barriers

Lack of structural availability was mentioned as a potential challenge by Davis et al. [24]. A significant barrier identified was the lack of dedicated clinical spaces and infrastructure necessary for effective multidisciplinary collaboration. This included insufficient access to specialized diagnostic equipment and facilities designed to support comprehensive care coordination.

### Relational connections

#### Facilitators

The concept of relational connections was discussed in all of the included studies (*n* = 7) as well as in the grey literature as a key facilitator to the success of a multidisciplinary clinic.

The intricacy of coordinating multidisciplinary inputs and ensuring that all team members are informed of and collaborate on each patient's diagnosis, care plan, and progress is crucial for the success of a multidisciplinary amyloidosis clinic [22–24, 26]. The importance of connections between many providers, but particularly between cardiologists, neurologists, hematologists, and nurse care coordinators, is highlighted as a key facilitator to fostering improved patient care and improved research programs [22, 24, 26, 32]. Hematologists work collaboratively within their own teams to plan and undertake procedures like stem cell transplants for patients with AL amyloidosis, demonstrating the importance of collaboration within specialties as well as between them [22]. At the Bulgarian Centre of Excellence (CoE), engagement between experts is stressed as a key facilitator to ensuring expert, tailored care is delivered [41].

Additionally, the embedding of most amyloidosis clinics within large academic centres supports essential connections to experts across fields, enhances collaborative care [32], and often leads to increased referrals [26]. The effectiveness of relational connections was noted to be bolstered by transparency about the roles and expertise of each team member, which is instrumental in fostering successful clinical outcomes [40].

### Communications

#### Facilitators

Communications play a critical role in healthcare delivery, particularly in multidisciplinary settings where complex diseases such as amyloidosis are managed. Five of the seven (71%) reviewed articles emphasize the importance of these communication practices as pivotal for advancing program objectives and care delivery.

Regular post-clinic and multidisciplinary meetings and communications between various departments, whether held in person or virtually and at varying intervals, are mentioned as crucial facilitators to the success of a multidisciplinary care approach. These meetings ensure that cohesive treatment plans, diagnoses, follow-up strategies, and program development initiatives are established and communicated among team members, enhancing the quality and continuity of patient care and the amyloidosis programs [22, 26, 32, 40, 41].

#### Barriers

Communication-related challenges were reported as barriers in two of the included articles (29%). Sperry et al. (2022) report scheduling constraints as a barrier to effective communication within the multidisciplinary team [22]. The challenge of coordinating regular meetings among expert providers with busy schedules can hinder the seamless exchange of information and collaboration that is necessary for advancing patient care management. Overcoming this barrier has involved moving the meeting to times when collaborators are more likely to be available (before the workday begins) and making use of virtual networks to facilitate the essential collaboration needed to efficiently support patient care.

The clinic in Greece reports an overall lack of communication and care coordination throughout Greece as hindering true collaboration as they look to create a more comprehensive, multidisciplinary amyloidosis pathway for care [23].

### Tension for change

#### Facilitators

Tension for change in relation to the development and implementation of multidisciplinary specialty care for amyloidosis was mentioned in all of the included articles (100%). Amyloidosis has been undertreated and underrecognized and care has not been well administered or coordinated in the past. The time to diagnosis has been quoted at 1–2 years [22, 26, 40], with a misdiagnosis rate of up to 57% [23]. There are now less invasive diagnostics and better treatment options, which has created a need for change [24, 41].

All of the included articles recognized the need for comprehensive care due to multisystem involvement, which necessitates subspecialty expertise across multiple disciplines for care and diagnosis [22–24, 26, 32, 40, 41].

### Mission alignment

#### Facilitators

Two of the seven articles (29%) discussed mission alignment as a facilitator.

Aligning the program goals with the overall mission and clinical operations of the organization was identified as an important facilitator to creating institutional buy-in [22, 26]. This alignment enhances patient care and supports the broader mission of institutions to deliver quality, innovation, and education in the medical field.

### Available resources

#### Facilitators

The construct of available resources was identified as a facilitator in four of the seven (57%) of the included articles as well as in the grey literature, encompassing funding, materials, space, and equipment. The potential for revenue generation from tests and referrals, especially in the United States context, presents promising funding prospects for clinics [22]. Specialty diagnostics such as 99 m technetium-pyrophosphate (PYP) scanning, SAP scintigraphy [42], cardiovascular imaging, and biopsy have significantly contributed to increased disease diagnosis. Additionally, access to the full range of therapeutic options and access to a genetics program are all key to diagnosis, management, and collaborative care [22, 24, 40, 41].

#### Barriers

Available resources are commonly cited as barriers, with funding constraints highlighted as a significant challenge (*n* = 5). The variability in healthcare funding structures [41] and overall funding-related challenges [24, 40] often result in insufficient support for establishing multidisciplinary care options. Additionally, the resource-intensive nature of amyloidosis care, necessitating almost twice the EMR entries compared to other heart failure patients, underscores the need for greater human and logistical resources. This, combined with the extensive coordination required due to multisystem involvement, stretches available resources further, complicating effective care delivery [22, 32].

### Leadership engagement

#### Facilitators

Leadership engagement was mentioned as a facilitator in 43% (*n* = 3) of the included articles.

Bumma et al. (2022); Davis et al. (2021); and Sperry et al. (2022) all cited active organizational and medical leadership engagement as a key to the continued development of the program and to ensure institutional buy-in [22, 24, 26].

#### Barriers

In 29% (*n* = 2) of the included articles challenges were reported related to leadership and institutional support in the context of securing funding and overall support for the program and the required time to be dedicated by staff [24, 32].

### Access to knowledge and information

#### Facilitators

Access to specialized knowledge and continuous education about amyloidosis is identified as a key in the grey literature. Access was also identified as a facilitator in the effective management of the disease, as highlighted across all seven articles (100%). The significance of having hematologists, cardiologists, and other providers with specialized training in amyloidosis is crucial for accurate diagnosis and treatment in a multidisciplinary setting [22, 26]. Continuous education and specific training to be able to recognize and treat the full range of amyloid subtypes foster excellence in care and the development of innovative strategies to enhance disease recognition [22, 41]. Fellowship programs and the incorporation of specialized training with the medical education curriculum support, equipping relevant clinicians with the expertise necessary for managing amyloidosis, ensuring high-quality patient care, facilitating program growth, and managing risk [20, 21, 29, 37, 41].

#### Barriers

The articles discussed Knowledge and information as barriers in 43% (*n* = 3). Apostolou et al. [23], Davis et al. [24], and Losada et al. [40] highlight the lack of specialty knowledge, often stemming from a lack of education in rare diseases, particularly amyloidosis, as a key barrier, impeding the ability to provide comprehensive care [23, 24, 40].

## Innovation domain

### Innovation evidence-base

#### Facilitators

The innovation evidence-based construct is recognized as a facilitator in the establishment of multidisciplinary clinics, as highlighted in three of the seven (43%) studies reviewed. Multidisciplinary care, validated across other rare and complex chronic diseases and by the UK Rare Disease Action Plan, is considered the gold standard in amyloidosis management. Multidisciplinary care offers superior patient outcomes through innovative treatment options, access to clinical trials, and comprehensive care coordination [23, 24, 26].

### Complexity

#### Facilitator

The complexity inherent in amyloidosis care, ranging from routine management to advanced therapies like stem cell transplants, is reported as a facilitator by one study within the American healthcare setting. In this case, the complexity highlights the importance of having a dedicated amyloidosis program for patient care as a unique opportunity for institutional revenue generation due to the complex interventions and cross-consultation required [22].

#### Barriers

The complexity of the diagnosis was mentioned in five of the included articles (71%) as a challenge to the establishment and operations of multidisciplinary clinics.

The complexity of amyloidosis, which is evident in its numerous clinical manifestations and systemic impact, is highlighted as a barrier as it demands extensive, tailored resourcing, longer patient visits, and additional physician time as compared to other diseases, making coordination and organization a challenge [22–24, 40, 41].

## Individual characteristics

### Innovation beneficiaries and subdomain: need

#### Facilitators

Within the domain of beneficiaries is the subdomain regarding the ‘need’ of the beneficiaries. While all (*n* = 7) mentioned these clinics' positive impact on patients, the implementation component of patient needs and resources was referenced in four of the seven articles (57%) and in the grey literature. Two articles emphasized the importance of patient involvement and engagement as pivotal for the success of these programs, with the Bulgarian-based clinic noting how patient participation aids in advancing research, especially in understanding genetic clusters within families [23, 41]. Additionally, Sperry et al. [22] and the NHS Commissioning Board [42] highlight the patient-reported need for more accessible care by citing the fact that some patients travel more than three hours to reach their local amyloidosis centre, underscoring a critical demand for more accessible amyloidosis care solutions [22, 42].

### Characteristics and subdomain: opportunity

#### Facilitators

The critical role of human resources was mentioned as a facilitator in all of the included articles (*n* = 7). Human resources, including the deployment of staff with expertise in amyloidosis and the incorporation of multidisciplinary roles, have been highlighted as facilitators in providing amyloidosis care. Nurses, nurse practitioners, pharmacists, and allied health professionals are essential in developing care pathways, patient education, and managing complex medication regimes. Dedicated support staff improve clinic efficiency and patient care by handling logistics and helping with research. Physicians who have knowledge, experience, and expertise in amyloidosis diagnosis and management and who are willing to come together to collaborate on care are the most critical facilitators in establishing these clinics [22–24, 26, 32, 40, 41].

#### Barriers

Two of the seven (29%) included articles highlighted human resources as a barrier. Skilled human resources are necessary for implementation, but they are challenging to find [24]. Once involved, the care for amyloidosis patients demands significant coordination and time, further straining these resources [22].

## Implementation process

### Planning

#### Facilitators

Three articles (43%) provided structured implementation plans for multidisciplinary amyloidosis care, outlining actionable steps to establish these centers. Sperry et al. [22] four-phase implementation strategy focusing on stakeholder engagement, resource allocation, staff training, and continuous quality improvement. Similarly, Losada et al. [40] adapt a comprehensive nine-step plan from strategies used in other rare disease care implementations. Nakov et al. [41] outline a five-step framework emphasizing early stakeholder involvement, training programs, and partnership development. See Table S5 for a comprehensive table of strategies and implementation plans proposed in the articles.

### Reflecting & evaluating

#### Facilitator

The process of reflecting and evaluating as a facilitator was highlighted in 57% (*n* = 4) of the included studies and in the grey literature. Sperry et al. [22] discussed the inclusion of regular multidisciplinary meetings that bring together staff and administrations to discuss program growth and evaluation. Davis et al. [24] and Nakov et al. [41] emphasize the importance of registry and database collection for the analysis of data that improves care and helps the sustainability and effectiveness of care delivery. Nativi-Nicolau et al. [32] found that cardiologists and amyloidosis nurses involved in amyloidosis clinics expressed wishes to grow their programs to improve education, research, and patient care, and extend community outreach and relationships. The NHS Commissioning Board [42] reports that the NHS prepares an annual report on diagnostic test turnaround times and has agreed upon quality-of-life measures for ongoing support of the program.

## Discussion

To our knowledge, this review represents the first of its kind in examining the implementation facilitators and barriers for implementing specialty multidisciplinary amyloidosis care. This review extends beyond the documented benefits of specialty clinics to include an understanding and description of the specific challenges and supports related to implementation and sustainability. The necessity for the implementation of amyloidosis specialty clinics and Centres of Excellence (CoEs) is driven by recent rapid advancements in diagnostics and therapeutics [8], the increasing prevalence of amyloidosis diagnosis globally and evolving technology to support such care [32, 43, 44]. These multidisciplinary clinics and centres, characterized by the collaborative delivery of expert care and resources, are critical in addressing the significant diagnostic and treatment delays and challenges faced by this growing patient population. Models for these types of programs in cancer care and similar types of diseases like sarcoidosis have shown significant benefits in improving patient outcomes through coordinated care efforts [34, 45–48].

### Facilitators

In extracting the data from the included articles and mapping them to the CFIR’s five domains, most facilitators were found within the inner setting domain of the CFIR.

Key facilitators in the inner setting include the provision of funding, space, and specialized equipment, crucial for the infrastructure of multidisciplinary amyloidosis care [24]. Particularly significant is the presence of knowledgeable human resources such as amyloidosis experts from cardiology, nephrology, neurology, and hematology, as well as nurses and nurse practitioners, who play a pivotal role in care coordination and education alongside ancillary and allied health staff, all of whom collaborate to provide comprehensive amyloidosis care [24, 26, 32, 41]. Similar facilitators are observed in cancer and sarcoidosis care, where multidisciplinary teams, that can include multiple specialists such as oncologists, radiologists, respirologists, and cardiologists, work together to provide comprehensive care [45–47]. These types of models of care for cancer and sarcoidosis management similarly benefit from dedicated funding, nurse coordinators, and specialized teams to manage the complex, multi-systemic nature of the disease [29, 46].

Technology underpins these efforts, enhancing communication and collaboration. Digital tools and platforms such as EMRs, virtual meetings, and patient registries facilitate the collection of comprehensive patient data, improve interaction and coordination of care among team members, and enable quality improvement initiatives [22–24, 26, 40].

Outside of the inner setting domain, key facilitators were noted in each of the other four domains of the CFIR. Importantly, within the outer setting domain, forming partnerships and relationships with patient advocacy groups and other amyloidosis centres is key to providing support, and education, and expanding the reach of these clinical settings. This connection with advocacy groups, pharmaceutical companies, and other centres has shown to be a key success factor in multidisciplinary clinics for cancer care and other complex diseases, providing the necessary support for patients in navigating a complex diagnosis [46, 48].

In the innovation domain, having strong evidence for the benefit of these clinics is highlighted as a key factor for access to funding and organisational support. Last, in the implementation and individual domains, the tremendous need of amyloidosis patients for enhanced education and clinical support points to the need for a collaborative model of care that acts as a driver for change and implementation [22–24, 26, 32, 40–42].

### Barriers

The primary barriers to the implementation of multidisciplinary amyloidosis clinics are spread across all five CFIR domains. The primary barriers stem from the complexity of the disease, limitations in available resources and institutional buy-in, and challenges related to coordinating care outside of the care setting.

The complexity of amyloidosis as a disease is characterized by its diverse clinical presentations and the multi-systemic impact of amyloid fibril deposition [20, 49]. The clinical manifestations and patient needs related to this disease are complex; this complexity necessitates the availability of multiple providers with expert knowledge and adaptability in clinical approaches, the availability of specialized diagnostic capabilities, and access to novel therapeutics. All of this, related to clinical complexity, demands extensive coordination and expertise [23, 40, 41]. Additionally, the lack of standardized guidelines and the difficulties in recruiting and retaining skilled multidisciplinary teams further complicate the establishment and operation of these specialized clinics [21]. Amyloid-specific education is not widely available, making it challenging to train and maintain staff education [40]. Similar barriers are noted in cancer and sarcoidosis care, where the need for multidisciplinary coordination and the recruitment of skilled teams are critical challenges [44–48].

Coordination between primary care providers and external referring sites for imaging and shared care has been highlighted as a key barrier due to limitations of knowledge and connectivity issues within the local healthcare system [32].

These challenges are further influenced by the broader healthcare system in which these centers operate, as funding models, referral structures, and institutional priorities vary widely between regions.

For example, the UK’s National Amyloidosis Centre operates under a centralized, government-funded model, allowing for consistent national referral pathways [50]. In contrast, the U.S. model often relies on institutional funding and private insurance revenue [22, 26]. Meanwhile, Canadian and European centers tend to blend public healthcare funding with academic partnerships and often rely on external funding for sustainability, which may impact the degree of multidisciplinary integration possible [23, 24, 40, 41]. These structural differences could shape how amyloidosis care centers are developed and sustained, affecting patient access, availability of multidisciplinary expertise, and long-term program maintenance. As a result, context-specific strategies are essential for successful implementation.

### Looking forward

Amyloidosis is a global issue, presenting significant challenges for both patients and providers across different healthcare settings [10, 43]. The nuances of barriers and facilitators underscore the complexity of establishing and operating amyloidosis clinics.

This review aims to inform the implementation of more comprehensive amyloidosis care by highlighting some implementation barriers and facilitators that can be leveraged in planning. However, this review also reveals substantial gaps in knowledge regarding the implementation process, as there were only a few articles found. None of the included studies undertook process evaluations or used implementation frameworks to describe the implementation of their clinics. Additionally, there was very little information included about country or system-specific challenges impacting implementation, and there was an overall lack of detailed reports on implementation successes, failures, and outcomes.

Given the complexity of implementing multidisciplinary clinics [19], process evaluations can provide valuable insights that may explain why the intervention has (or has not) been implemented as intended and how different contextual factors may have influenced overall intervention outcomes [51]. Despite these challenges, several strategies are emerging to grow and facilitate multidisciplinary amyloidosis care. Other comprehensive care programs for cancer, sarcoidosis, and other complex conditions have reported using structured care pathways and telemedicine to enhance coordination and connectivity among care providers [27, 36, 45, 46]. Additionally, international efforts to standardize multidisciplinary amyloidosis care are increasing, complementing these strategies. Organizations such as the International Amyloidosis Society and various professional national associations play a critical role in facilitating knowledge transfer and reducing barriers to implementation by developing guidelines, setting standard nomenclature, hosting international conferences, and supporting fellowship programs [20, 40, 52]. Such initiatives are crucial for addressing the lack of standardized care pathways and ensuring access to high-quality amyloidosis care across different healthcare settings.

By highlighting these facilitators and barriers, this scoping review contributes to a better understanding of the critical factors influencing the successful implementation of multidisciplinary amyloidosis clinics. With this understanding of the implementation barriers and facilitators, healthcare administrators and policymakers can better allocate resources to support multidisciplinary amyloidosis care, ensuring that they are directed towards areas that will leverage the facilitators and mitigate barriers to improve amyloidosis outcomes. Additionally, clinicians and implementation teams can leverage this understanding in multidisciplinary clinics' design, implementation, and sustainability planning.

### Strengths and limitations

The review has several strengths. First, the search strategy included several databases and snowball searching for relevant articles. Second, the review was guided by expert researchers with advanced knowledge of the theoretical model, the scoping review process, and multidisciplinary care in amyloidosis.

Limitations to this study include the lack of use of implementation outcome terminologies as well as a lack of theoretical implementation frameworks used in reporting across the literature and some degree of overlap among constructs. This required the interpretation of findings based on operational definitions in the CFIR. Although this may have resulted in the misclassification of some findings, they were applied as closely as possible to the CFIR’s operational definitions. While no quality assessment was conducted, the included studies were primarily reports of clinics, which often lacked rigorous methodological approaches. This limitation may impact the generalizability and strength of the findings. Future research would benefit from including more methodologically rigorous studies to strengthen the evidence base for the implementation of multidisciplinary amyloidosis care. Our search was limited to studies in English that were electronically available. This is likely a limitation, as amyloidosis clinics exist globally and it is possible that key implementation papers exist describing clinics that are only available in other languages. Last, the timeframe for included studies (2013–2023) limits insights into the possibility of the more recent establishment of amyloidosis centers following Tafamidis approval and the expansion of scintigraphy as a diagnostic tool [20, 21]. Future research is needed to capture the evolving landscape of specialty care.

## Conclusion

The seven articles synthesized in this scoping review present the facilitators and barriers that impact the execution of multidisciplinary specialty amyloidosis clinics globally. The review findings have implications for clinical leaders and decision-makers interested in implementing amyloidosis clinics to identify common facilitators and barriers to implementation and appropriately allocate resources. Moreover, clinicians and implementation teams can learn from the barriers and facilitators in other clinics and apply the learnings to implementation initiatives in their own contexts. Importantly, the findings of this review highlight a significant gap in the literature surrounding the operation of these necessary and complex care settings, demonstrating a need for further research in this area.

## Supplementary Information

Supplementary Material 1.

Supplementary Material 2.

## Data Availability

The datasets used during the current study are available from the corresponding author on reasonable request.

## Abbreviations

DN: Doctor of Nursing
AL: Light chain amyloidosis
ATTR or TTR: Transthyretin amyloidosis
HFpEF: Heart Failure with Preserved Ejection Fraction
CFIR: Consolidated Framework for Implementation Research
JBI: Joanna Briggs Institute
NHS: National Health Service
IT: Information Technology
CoE: Centre of Excellence
PYP: Pyrophosphate
