# Supplementary material for: Integrated specialty care for amyloidosis: a scoping review using the Consolidated Framework for Implementation Research
Source: BMC Health Serv Res. 2025 Mar 21;25:415. doi: 10.1186/s12913-025-12520-3 (PMC11927205; doi:10.1186/s12913-025-12520-3)
Supplement: Supplementary file 1 — Supplementary Material 1. [file 12913_2025_12520_MOESM1_ESM.docx]

**Supplementary Materials**

**Supplementary Material Description**

Table S1 – Search strategy for included academic electronic databases

Table S2 – Overview of included academic literature

Table S3 -  Overview of included grey literature

Table S4 – Outline of included academic literature and respective facilitators and barriers by CFIR construct

Table S5 - Overview of Implementation Strategies

**Supplementary Materials**

**Supplementary Table S1  - Academic Literature Search Strategy for Electronic Databases**

**OVID Medline**

| *1.* | *(amyloid* or "cardiac amyloidosis" or "light chain amyloidosis" or "transthyretin amyloidosis" or "AL amyloidosis" or transthyretin or "protein misfolding disease" or "hereditary amyloidosis" or "systemic amyloidosis" or " amyloid deposits" or wATTR or ATTR or hATTR or "hematological malignanc*").tw,kf.* |
| --- | --- |
| *2.* | *((multidisciplin* or integrat* or holistic* or multiprofession* or comprehensive or collaborat* or interdisciplin* or inter-disciplin* or multi-disciplin* or multi-profession* or special*) adj5 (care or team* or health* or communication)).tw,kf.* |
| *3.* | *((cent* adj2 excellence) or "special* clinic*" or "clinical path*" or "critical path*" or "optimal clinical care setting*" or "guideline*" or "position statement*").tw,kf.* |
| *4.* | *exp Amyloid Neuropathies, Familial/* |
| *5.* | *exp Amyloidosis, Familial/ or amyloidosis.mp. or exp Amyloidosis/ or exp Immunoglobulin Light-chain Amyloidosis/* |
| *6.* | *exp Patient Care Team/* |
| *7.* | *exp Comprehensive Health Care/ or Critical Pathways/* |
| *8.* | *1 or 4 or 5* |
| *9.* | *2 or 3 or 6 or 7* |
| *10.* | *8 and 9* |
| *11.* | *limit 10 to (english language and yr="2008 -Current")* |
| *12.* | *(Animals/ or Models, Animal/ or Disease Models, Animal/) not Humans/* |
| *13.* | *((animal or animals or canine* or dog or dogs or feline or hamster* or lamb or lambs or mice or monkey or monkeys or mouse or murine or pig or pigs or piglet* or porcine or primate* or rabbit* or rats or rat or rodent* or sheep* or veterinar*) not (human* or patient*)).ti,kf,jw.* |
| *14.* | *12 or 13* |
| *15.* | *11 not 14* |

**Ovid EMBASE**

| 1. | exp familial amyloidosis/ or exp AL amyloidosis/ or exp heart amyloidosis/ or exp ATTR amyloidosis/ or exp amyloidosis/ |
| --- | --- |
| 2. | (amyloid* or "cardiac amyloidosis" or "light chain amyloidosis" or "transthyretin amyloidosis" or "AL amyloidosis" or transthyretin or "protein misfolding disease" or "hereditary amyloidosis" or "systemic amyloidosis" or " amyloid deposits" or wATTR or ATTR or hATTR or "hematological malignanc*").tw,kf. |
| 3. | ((multidisciplin* or integrat* or holistic* or multiprofession* or comprehensive or collaborat* or interdisciplin* or inter-disciplin* or multi-disciplin* or multi-profession* or special*) adj5 (care or team* or health* or communication)).tw,kf. |
| 4. | ((cent* adj2 excellence) or "special* clinic*" or "clinical path*" or "critical path*" or "optimal clinical care setting*" or "guideline*" or "position statement*").tw,kf. |
| 5. | exp multidisciplinary team/ |
| 6. | exp interdisciplinary communication/ or clinical pathway/ |
| 7. | 3 or 4 or 5 or 6 |
| 8. | 1 or 2 |
| 9. | 7 and 8 |
| 10. | limit 9 to yr="2008 -Current" |
| 11. | limit 10 to english language |
| 12. | (exp animal/ or nonhuman/) not exp human/ |
| 13. | 11 not 12 |

**CINAHL**

1. (MH "Amyloidosis, Familial") OR (MH "Amyloidosis, Cardiac") OR (MH "Amyloidosis")
2. (MH "amyloid*" or MH "cardiac amyloidosis" or MH "light chain amyloidosis" or "transthyretin amyloidosis" or "AL amyloidosis" or "transthyretin" or MH "protein misfolding disease" or MH "hereditary amyloidosis" or MH "systemic amyloidosis" or MH "amyloid deposits" or MH "wATTR" or MH "ATTR" or MH "hematological malignanc*")
3. (MH "Multidisciplinary Care Team")
4. (MH "multidisciplin*" or MH "multidisciplinary clinic" or MH "multidisciplinary communication" or MH "multidisciplinary care team" or MH "multidisciplinary care team*" or MH "interdisciplinary*" or MH "comprehensive*" or MH "integrat*" or MH "holistic")
5. (MH "Multidisciplinary Care Team")
6. (MH "Excellence")
7. S1 OR S2
8. S3 OR S4 OR S5 OR S6
9. S7 AND S8
10. S7 AND S8

**Supplementary Table S2  - Overview of Included Academic Literature**

| ***Authors and Dates*** | ***Title*** | ***Aim*** | ***Design*** | ***Location*** |
| --- | --- | --- | --- | --- |
| Apostolou et al, 2023 | The patient pathway in ATTR-CM in Greece and how to improve it: A multidisciplinary perspective | To identify and lay out the complexities of ATTR-CM disease management, provide an expert perspective regarding the Greek healthcare setting, and outline practical recommendations on how to improve the patient journey. | Qualitative | Greece |
| Bumma et al, 2022 | Multidisciplinary amyloidosis care in the era of personalized medicine | To highlight the value of a multidisciplinary comprehensive amyloidosis clinic using the experience of The Ohio State University. | Qualitative | Ohio, United States of America |
| Davis et al, 2021 | Establishing a Cardiac Amyloidosis Clinic: A Practical Primer for Cardiologists | To report on barriers to program development and key components of a best-practice Cardiac Amyloidosis Program as reported through consensus. | Qualitative | Canada |
| Lousada et al, 2020 | Multidisciplinary approach in the management of hATTR | Discussion about benefits, objectives, and the experience of a multidisciplinary TTR team in Spain. | Qualitative | Palma, Spain |
| Nakov et al, 2020 | Transthyretin amyloidosis: Testing strategies and model for center of excellence support. | To focus on the importance of performing the most appropriate testing strategies for ATTR amyloidosis and establishing a CoE for this rare disease. | Qualitative | Sofia, Bulgaria |
| Nativi-Nicolau, 2021 | Best Practices in Specialized Amyloidosis Centers in the United States: A Survey of Cardiologists, Nurses, Patients, and Patient Advocates | To gain insight into the best practices and unique characteristics of US amyloidosis centers. | Qualitative | United States of America |
| Sperry et al, 2022 | Comprehensive approach to cardiac amyloidosis care: considerations in starting an amyloidosis program. | To describe considerations and steps in starting and growing an amyloidosis program given the increase in disease awareness and recognition. | Qualitative | Kansas & Phoenix, United States of America |

**Supplementary Table S3  - Overview of Included Grey Literature**

| National Health Service, England, 2013/14 | NHS Standard Contract for Diagnostic Service for Amyloidosis | Provide an overview of the national diagnostic and management service for patients with amyloidosis and inherited periodic fever syndromes. | Contract | England |
| --- | --- | --- | --- | --- |
| Fajardo, Nativi-Nicolau, Di Paulo, 2022 | Developing an Amyloidosis Center of Excellence: Path to Success and Overcoming Challenges | To identify strategies, steps, and challenges of developing a centre of excellence for amyloidosis. | Presentation at Amercian Heart Association, presentation of academic article included in review (Nativi-Nicolau et al., 2021). | United States of America |
| Sperry et al, 2022 | Quality of Life Considerations and Multidisciplinary Team-Based Care for Patients with hATTR Amyloidosis | To present on ATTR amyloidosis and on multidisciplinary care in an amyloidosis center. | Presentation at American Heart Association, presentation of academic article included in review (Sperry et al., 2022) | United States of America |

**Supplementary Table S4 -** *Academic Literature Barriers and Facilitators by CFIR Domain*

| **CFIR Domains** | **Outer Setting** | | **Inner Setting** | | | | | | | | **Innovation** | | **Individual** | | **Implementation Process** | |
| --- | --- | --- | --- | --- | --- | --- | --- | --- | --- | --- | --- | --- | --- | --- | --- | --- |
| CFIR construct | **Policies and laws** | **Partnerships and connections** | **Structural Characteristics** | **Relational Connections** | **Communications** | **Tension for Change** | **Mission Alignment** | **Available Resources** | **Leadership Engagement** | **Access to Knowledge and Information** | **Evidence Base** | **Complexity** | **Innovation Beneficiaries: Need** | **Characteristics: Opportunity** | **Planning** | **Reflecting and Evaluating** |
| Apostolou et al, 2023 | F | F | F | F | F/B | F |  |  |  | F/B | F | B | F | F |  |  |
| Bumma et al, 2022 | F | F | F | F |  | F | F |  | F | F | F |  | F | F |  |  |
| Davis et al, 2021 | F | F/B | F/B | F |  | F |  | F/B | F/B | F/B | F | B | F | F/B |  | F |
| Losada et al, 2020 | F/B | F |  | F | F | F |  | F/B |  | F/B |  | B | F | F | F |  |
| Nakov et al, 2020 | B | F/B |  | F | F | F |  | F/B |  | F |  | B | F | F | F | F |
| Nativi-Nicolau, 2021 |  | F/B | F | F | F | F |  | B | B | F |  |  | F | F |  | F |
| Sperry et al, 2022 |  | F/B | F | F | F/B | F | F | F/B | F | F |  | F/B | F | F/B | F | F |

**Supplementary Table S5 -** *Implementation Strategies*

| **Publication** | **Location** | **Achieving Multidisciplinary Care** |
| --- | --- | --- |
| Sperry et al, 2022 | United States of America | 1. Identify multidisciplinary stakeholders 2. Develop overarching program goals 3. Create institutional buy-in 4. Emphasize program growth and development |
| Losada et al, 2020 | Spain | 1. Define the problem 2. Decide on goals 3. Gather information 4. Seek opinions 5. Discuss and expand the problem 6. Develop potential solutions 7. Share opinions 8. Evaluate potential solutions and choose the best one 9. Summarize the plan and agree on the distribution of tasks across team members |
| Nakov et al, 2020 | Bulgaria | 1. Establish a dedicated team of multidisciplinary experts 2. Engage with patient advocacy groups 3. Initiate a specific training regime to continue education for new and 4. existing members of the team 5. Source appropriate funding to ensure sustainability 6. Schedule regular team meetings to ensure an individual plan for patient diagnosis, treatment and follow-up |
